# Influence of Dietary Calcium Intake on Skeletal Health and Body Composition in an Italian Elderly Population

**DOI:** 10.3390/nu17132073

**Published:** 2025-06-21

**Authors:** Carla Caffarelli, Antonella Al Refaie, Caterina Mondillo, Guido Cavati, Anna Lora, Luigi Gennari, Ranuccio Nuti, Stefano Gonnelli

**Affiliations:** 1 Division of Internal Medicine, Department of Medicine, Surgery and Neuroscience, University of Siena, 53100 Siena, Italy; antonellaalrefaie@gmail.com (A.A.R.); caterinamondillo28@gmail.com (C.M.); guido.cavati@student.unisi.it (G.C.); anna.lora@student.unisi.it (A.L.); luigi.gennari@unisi.it (L.G.); ranuccio.nuti@unisi.it (R.N.); stefano.gonnelli@unisi.it (S.G.); 2 Division of Internal Medicine I, San Giuseppe Hospital, 50053 Empoli, Italy

**Keywords:** dietary calcium intake, bone mineral density, bone fractures, lean mass, fat mass

## Abstract

**Background:** Calcium is the most abundant mineral in the human body and is essential not only for bone health but also for many other physiological functions. In fact, dietary calcium intake is important not only for bone health but also for fat mass and overall body composition. This study aimed to evaluate the potential effects of dietary calcium intake on bone mineral density (BMD), body composition, and fragility fractures. **Methods:** In a cohort of 173 consecutive elderly men and 939 women aged 55 and over, living in Siena, Italy, daily dietary calcium intake was evaluated using a food frequency questionnaire (FFQ) specifically validated for the Italian population. Bone mineral density at various skeletal sites and body composition were measured in all participants using a Lunar Prodigy densitometer. Additionally, the serum levels of vitamin D and bone turnover markers were assessed, and the presence of prevalent atraumatic fractures was documented. **Results:** Across all age groups, calcium intake was consistently higher in males (898.40 ± 312.87 mg/day) than in females (821.95 ± 351.3 mg/day); the prevalence of subjects in the lowest tertile of calcium intake was significantly higher among females than males (31.4% vs. 14.5% *p* < 0.05). Moreover, dietary calcium intake showed an inverse relationship with body fat mass in women (*p* < 0.05) and a positive association with lean mass in men (*p* < 0.05). Two hundred twenty-eight (24.3%) women and forty-eight (27.8%) men had a history of one or more fractures, and in both sexes, subjects with prevalent fractures had significantly lower dietary calcium intake values than those without fractures. **Conclusions:** This study indicates that inadequate calcium intake remains widespread in the Italian population, especially among subjects with low BMD and a history of fractures. Furthermore, this study confirms that dietary calcium intake significantly affects body composition.

## 1. Introduction

Calcium is the most abundant mineral in the human body and is essential for numerous physiological processes, including cardiac function, bone health, muscle function, and enzymatic signaling in biochemical pathways [1]. Approximately 99% of the body’s calcium is stored in the skeleton, primarily in the form of crystals, while plasma contains only 1% of the total body calcium. Blood calcium levels must be maintained within a narrow range of 8.8–10.4 mg/dL. Several hormones, including parathyroid hormone (PTH), calcitonin, and calcitriol, play crucial roles in maintaining calcium homeostasis [1,2,3].

It is widely recognized that attaining an adequate peak bone mass—a crucial factor in preventing age-related bone loss—is significantly influenced by calcium intake [4]. Calcium absorption efficiency plays a vital role in maintaining calcium balance. However, calcium absorption tends to decline with age, a trend that becomes more pronounced in women during menopause [5,6]. Several factors contribute to inefficient calcium absorption or malabsorption in the gastrointestinal tract, including reduced renal synthesis of 1,25-dihydroxyvitamin D, a decline in intestinal vitamin D receptors, resistance to 1,25-dihydroxyvitamin D, deficiency of 25-hydroxyvitamin D, and decreased active calcium transport [6]. Moreover, low calcium intake influences bone metabolism and bone markers and is associated with an increased risk of fractures [7,8]. While calcium is well known for its role in bone health, it also plays a significant role in muscle function, fat metabolism, and overall body composition [9]. In particular, in muscle cells, the contraction and relaxation of myosin fibers, as well as glycolytic and mitochondrial metabolism, are regulated by calcium levels [10]. Studies have demonstrated a link between higher calcium intake and a reduced risk of sarcopenia in older adults, suggesting a potential protective effect of calcium on muscle mass. Additionally, higher calcium intake may be associated with greater muscle strength and a lower body fat percentage [11].

Daily calcium requirements vary throughout the lifespan [6,12]. Global calcium consumption patterns differ significantly, influenced by a complex interplay of dietary factors, cultural practices, and socioeconomic status. Moreover, recent studies suggest that variations in dietary protein intake can significantly affect intestinal calcium absorption. In fact, dual stable isotope studies have shown that higher dietary protein levels are associated with a significant increase in intestinal calcium absorption [13]. The recommended dietary allowance for calcium differs across various national guidelines. Additionally, ethnic variations in fractional calcium absorption have been observed. Dual isotope studies have shown that white postmenopausal women have significantly lower fractional calcium absorption compared to Hispanic and Black women [14]. Among Chinese adults, the average fractional calcium absorption has been reported to be 33% with a daily calcium intake of 600 mg [15]. The higher fractional absorption and retention of calcium observed in Asian Chinese individuals may represent a physiological adaptation to their characteristically low calcium intake [15]. The associations between dietary calcium intake and bone health have been examined in numerous studies, including several in Italy; however, these investigations often involved heterogeneous populations and have yet to yield robust conclusions regarding the relationships between calcium intake and bone mineral density (BMD), body composition, and fragility fractures.

This study aimed to evaluate dietary calcium intake and its potential correlation with BMD, body composition, and fragility fractures in a representative sample of the adult outpatient population in the city of Siena, Italy. In particular, a key novelty of this study is the investigation into the dietary calcium intake and body composition.

## 2. Materials and Methods

### 2.1. Sample and Ethics

This single-center study assessed bone status in a cohort of elderly individuals residing in Siena, Italy. The source population included 173 consecutive men and 639 women aged 55 and older who participated in the larger Siena Osteoporosis Study, an epidemiological study conducted between July 2004 and January 2008 in collaboration with general practitioners. This study aimed to evaluate risk factors for metabolic bone diseases in Siena’s elderly population, which comprises approximately 60,000 inhabitants. Subjects for the epidemiological survey were recruited by general practitioners who, starting from a specific date, invited the first 50 consecutive ambulatory postmenopausal women and the first 25 consecutive men aged 55 years or older, not treated with anti-osteoporotic drugs or calcium supplements and without evidence of cancer or any relevant diseases, to participate in the study [16]. This study was conducted in accordance with the 1964 Helsinki Declaration and its later amendments. Moreover, the research protocol received approval from the Ethics Committee of Siena University Hospital (ID-12715-20). Written informed consent was obtained from all participants. Prior to statistical analysis, all collected data underwent anonymization procedures.

### 2.2. Dietary Assessment

For all subjects, a detailed medical history was obtained. Daily dietary calcium intake was evaluated for each participant using a food frequency questionnaire (FFQ) specifically validated for the Italian population [17]. The daily calcium intake was calculated based on the responses to the FFQ. The questionnaire was administered in conjunction with the bone densitometry appointment. Participants who were using calcium supplements or medications that interfere with calcium metabolism at the time of enrollment were excluded from this study. In addition, height and weight were measured in a standardized fashion. The body mass index (BMI) was calculated as weight in kilograms divided by the square of height in meters.

### 2.3. Bone Mineral Density, Body Composition, Fractures, and Laboratory Tests

Bone mineral density was measured in all participants at the lumbar spine [LS-BMD], femoral subregions (femoral neck [FN-BMD] and total hip [TH-BMD]), and total body [WB-BMD], using dual-energy X-ray absorptiometry (DXA) (Lunar Prodigy; GE Healthcare, Waukesha, WI, USA). Diagnostic criteria for osteoporosis and osteopenia were based on World Health Organization (WHO) definitions: osteoporosis was defined as a T-score of −2.5 or lower, while osteopenia (low bone density) was defined as a T-score between −1.0 and −2.5. T-scores were calculated using sex-matched Italian reference data. Fat mass (FM) and lean mass (LM) were also determined using the same DXA device (Lunar Prodigy; GE Healthcare, Waukesha, WI, USA) in conjunction with Encore 2002 software. Moreover, the presence of prevalent atraumatic fractures was recorded, and all reported fractures were physician-adjudicated based on radiology reports. Fractures involving the patella, toes, fingers, skull, and face were not considered.

Following an overnight fast, blood samples were collected from all participants between 08:00 and 09:00 a.m. Serum samples were subsequently stored at −70 °C until analysis. The analytes measured included calcium (Ca), phosphate (P), creatinine (Cr), alkaline phosphatase (ALP), albumin (Alb), 25-hydroxyvitamin D (25OHD), parathyroid hormone (PTH), bone alkaline phosphatase (B-ALP), and type I collagen β-carboxy-telopeptide (β-CTX). Serum 25OHD concentrations were measured using a radioimmunoassay (DiaSorin, Saluggia, Italy), demonstrating intra- and inter-assay coefficients of variation of 6.8% and 9.2%, respectively. Serum PTH was assessed by an immunoradiometric assay (Total Intact PTH Antibodies Lab. Inc.; Santee, CA, USA), and the intra- and inter-assay coefficients of variation were 3.6 and 4.9%, respectively. Serum B-ALP concentrations were quantified using a chemiluminescent immunoassay (LIAISON BAP Ostase, DiaSorin Inc., Stillwater, MN, USA) exhibiting intra- and inter-assay coefficients of variation of 4.2% and 7.9%, respectively. Serum β-CTX levels were determined via enzyme-linked immunosorbent assay (Serum Cross-Laps ELISA; Nordic Bioscience Diagnostics, Herlev, Denmark), with intra- and inter-assay coefficients of variation of 5.4% and 7.9%, respectively. The measurement of all the other lab parameters was carried out with a colorimetric method (Autoanalyzer, Falcor 350 Menarini, Florence, Italy).

### 2.4. Statistical Analysis

All outcomes and predictor variables were continuous and described as means ± standard deviations (SDs). Clinical data and initial values of the measured variables in the study groups were compared using Student’s *t*-test for unpaired data. Categorical variables were subjected to comparison using the Chi-square test. All tests were two-sided, and *p* < 0.05 was considered statistically significant. Associations between different parameters were examined through Pearson’s correlation or Spearman’s correlation, as appropriate, or via partial correlation analysis. For the regression, the coefficients (β-coefficients) and their 95% confidence intervals (CIs) are described. The adjusted R^2^ value is also given. The stepwise automatic procedure for selecting the models was used to avoid the occurrence of misleading findings due to highly dependent correlated variables. The collinearity diagnostics confirmed that there was no variance inflation factor greater than 1.5. However, since some predictors might be affected by age, multiple regression analysis was performed with or without age. The results were similar. The data were analyzed with the SPSS statistical package for Windows version 16.0 (SPSS Inc., Chicago, IL, USA).

## 3. Results

Table 1 shows the clinical, biochemical, and densitometric characteristics of the study participants divided by gender. As expected, all densitometric parameters were higher in men than in women. Moreover, vitamin D levels were significantly higher in men, while PTH levels were significantly lower compared to women. Conversely, both bone turnover markers, B-ALP and β-CTX, were higher in women than in men.

**Figure 1:**
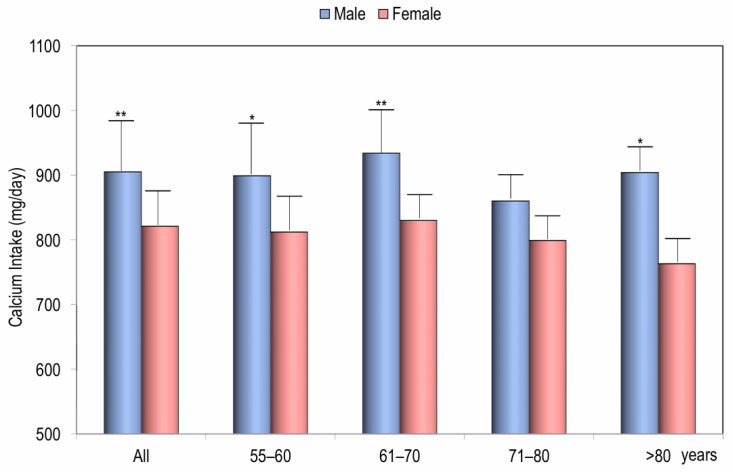
Calcium intake in all males and females and according to the decade of age. * *p* < 0.05, ** *p* < 0.01 for Student’s *t*-test of males vs. females.

The values of daily calcium intake, as assessed by a validated food frequency questionnaire, ranged from 271 mg/day to 2250 mg/day. The mean calcium intake was 821.95 ± 351.3 mg/day for females and 898.40 ± 312.87 mg/day for males, with a statistically significant difference (*p* < 0.01) (Figure 1). Figure 1 also illustrates the daily dietary calcium intake of the study population, categorized by age decade and gender. Across all age decades, calcium intake was consistently higher in males than in females, with the most significant difference observed in the sixth decade (*p* < 0.05).

**Figure 2:**
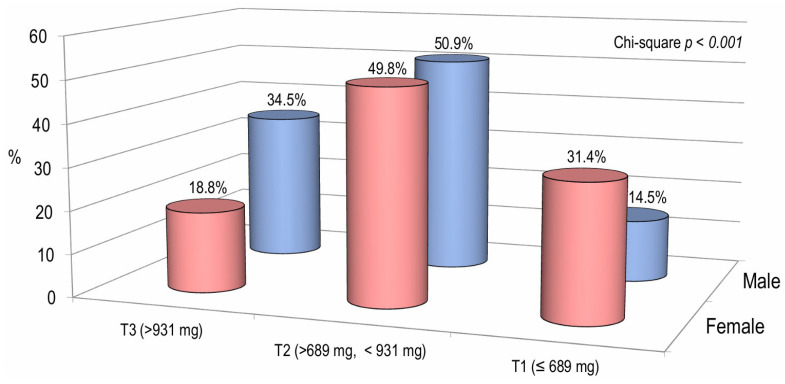
The proportion of patients categorized by tertiles of calcium intake according to gender.

Figure 2 illustrates the percentage of male and female subjects categorized by tertiles of calcium intake. The prevalence in the lower tertile of calcium intake was significantly higher in females than in males (31.4% vs. 14.5%, respectively). Instead, in the highest tertile of calcium intake, the prevalence of males was significantly higher than that of females (34.5% versus 18.8%, respectively) (Figure 2).

In both sexes, BMD values at all skeletal sites tended to be higher in the tertiles with greater calcium intake, but statistical significance (*p* < 0.05) was reached only for WB-BMD in males and FN-BMD in females. The age- and BMI-adjusted partial correlations between calcium intake and 25OHD, PTH, B-ALP, and β-CTX in both male and female subjects are presented in Table 2. In both men and women, 25OHD showed a positive correlation with calcium intake; however, this association reached statistical significance only in the female group (*p* < 0.01). Conversely, the correlations of calcium intake with PTH, B-ALP, and β-CTX were negative, with statistical significance observed for PTH and β-CTX in women and only for β-CTX in men (*p* < 0.05).

**Figure 3:**
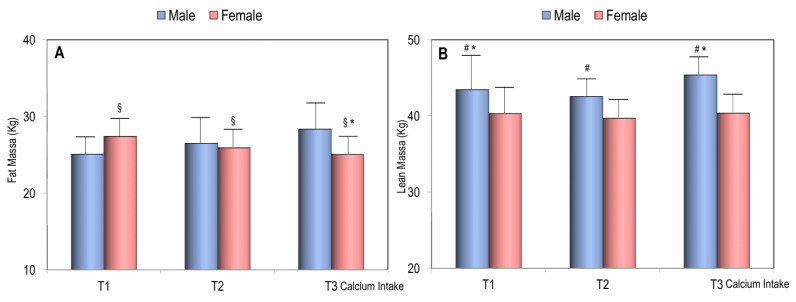
Fat mass (**A**) and lean mass (**B**) in males and females according to the tertiles of calcium intake. * *p* < 0.05 for Student’s *t*-test of males vs. females; ^§^ *p* < 0.05 for ANOVA test according to the calcium intake tertiles in females; ^#^ *p* < 0.05 for ANOVA test according to the calcium intake tertiles in males.

Figure 3 shows the values of fat mass and lean mass in men and women across different tertiles of dietary calcium intake. In both men and women, fat mass was higher in individuals with lower calcium intake compared to those in the highest tertile of calcium intake. However, while fat mass significantly decreased with increasing calcium intake in females, it remained stable across tertiles in males (Figure 3). As expected, lean mass was consistently higher in men than in women. However, while lean mass significantly increased with higher calcium intake in men (*p* < 0.05), it remained stable across the tertiles in women (Figure 3).

**Figure 4:**
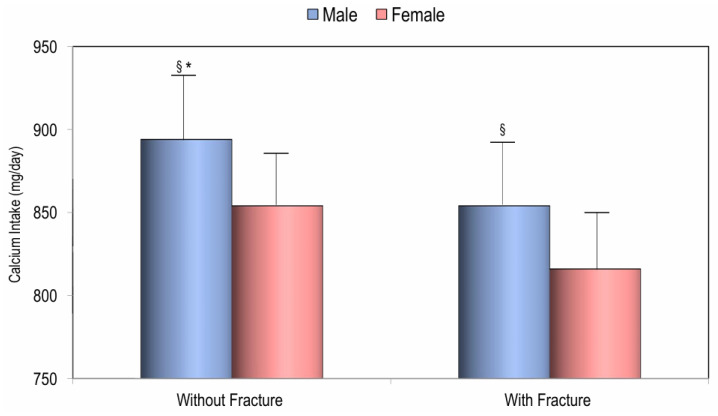
Calcium intake in males and females according to history of fractures. * *p* < 0.05 for Student’s *t*-test of males vs. females; ^§^ *p* < 0.05 for Student’s *t*-test without fractures vs. with fractures.

Two hundred twenty-eight (24.3%) women and 48 (27.8%) men had a history of one or more fractures, and in both sexes, subjects with prevalent fractures had significantly lower dietary calcium intake values than those without fractures (Figure 4).

Multivariate models were used to examine the relationships between BMI, age, B-ALP, 25OHD, PTH, β-CTX, calcium intake, and history of fracture with BMD at the lumbar spine, proximal femur, and whole body in males and females separately (Table 3). In males, BMD at all sites was positively associated with BMI. Additionally, positive associations were observed between calcium intake and BMD at the femoral neck, the total hip, and the whole body (Table 3). In females, we observed a positive association between BMI and BMD at all skeletal sites, while age was negatively associated with BMD at the lumbar spine, proximal hip, and whole body. Furthermore, a history of fractures was negatively associated with lumbar BMD and WB-BMD, whereas B-ALP was negatively associated with total TH-BMD and WB-BMD. Finally, in the female population, calcium intake was positively associated only with WB-BMD (Table 3).

## 4. Discussion

The mean daily calcium intake in the present study (898.4 mg in males and 821.9 mg in females), although slightly higher than in other Italian studies [18,19,20], remains significantly lower than the levels recommended by most Italian and international guidelines, which consider an intake of at least 1000–1200 mg adequate for the adult population [21,22,23]. In this single-center study, consistent with findings from other Italian studies, we observed that women have a significantly lower calcium intake than men, with intake levels tending to decrease with age [18]. This is a critical concern, as intestinal calcium absorption progressively declines with age, and the combination of these factors may increase the risk of fragility fractures in older women [1,6].

Another interesting finding of this study was that, in women, fat mass significantly decreased with increasing calcium intake. Several epidemiological cross-sectional studies have confirmed the inverse association between a diet rich in calcium and total body fat [24,25].

These latter studies have reported that the anti-obesity and anti-fat mass mechanisms of calcium intake may be related to a high intracellular calcium concentration, which attenuates adipocyte lipid accretion and stimulates lipolysis, promoting greater rates of fat oxidation [11]. Furthermore, dietary calcium intake may affect fat metabolism. Specifically, calcium can bind with fatty acids in the intestine to form insoluble soaps, which enhance fecal fat excretion and reduce fat absorption [26,27]. Furthermore, a recent Cochrane systematic review, aiming to assess the effects of calcium supplementation on weight loss in individuals living with overweight or obesity, reported that calcium supplementation may result in a small reduction in BMI, waist circumference, and fat mass. However, the evidence for this relationship was of low to moderate certainty [28]. In fact, in a study of overweight and obese men and women on an energy-restricted diet, 15 weeks of a high-calcium regimen did not produce greater weight or fat loss than a low-calcium regimen [29]. Recently, some authors have suggested that the effect of calcium intake on fat mass may be mediated by its influence on muscle mass and strength [11]. In particular, a recent study reported that, in physically active Chinese individuals, calcium intake in relation to phosphorus was negatively associated with visceral fat and positively associated with higher skeletal muscle mass [30]. Other studies have confirmed that increased dietary calcium intake is linked to positive effects on muscle mass [31,32].

In our study, the greater lean mass in the tertile with the highest dietary calcium intake was found to be significantly greater in males but not in females. At the moment, we do not have a convincing explanation for this difference. However, it can be hypothesized that males with a higher calcium intake may have a more active lifestyle than females.

In our study, both males and females showed slightly lower lumbar and femoral BMD values in the lowest calcium intake tertile compared to the highest intake tertile, though the difference was far from statistically significant. However, in the stepwise regression model, calcium intake was identified as a predictor of FN-BMD, TH-BMD, and WB-BMD in males and WB-BMD in females. Indeed, the literature presents conflicting data on the relationship between calcium intake and BMD. A cross-sectional study utilizing data from the 2008–2010 Korea National Health and Nutrition Examination Survey (KNHANES), involving approximately 7000 individuals over the age of 50, found that a calcium intake of less than 400 mg/day was linked to lower BMD [33]. Conversely, a calcium intake exceeding 1200 mg/day showed a positive correlation with BMD [33]. However, the relationship between calcium intake and BMD was not consistently linear, and adequate vitamin D levels appeared to mitigate the adverse effects of low calcium intake on bone health [33]. A study conducted on 4958 community-dwelling women from the U.S. NHANES III population-based survey found that calcium intake had a significant positive association with femoral neck BMD in women with serum 25OHD concentrations below 50 nmol/L but not in those with higher concentrations; therefore, the study concluded that an increased calcium intake was beneficial only for women with 25OHD levels below 50 nmol/L [34]. Partially conflicting, the meta-analysis by Tai and colleagues (which included 59 studies) found that increasing calcium intake from dietary sources raised femoral BMD by 0.6–1.0% and femoral and spinal BMD by 0.7–1.8% at two years. However, the increase in BMD at later time points remained similar to that observed at one year [35]. Moreover, two Italian studies conducted on populations with a dietary calcium intake that was not particularly low did not find any relationship between calcium intake and BMD values at various skeletal sites [19,20]. Finally, in a study conducted on individuals aged 70 and older, Andersen et al. reported that a habitual high calcium intake exceeding the recommended dietary allowance in elderly women and men provided no benefits for hip or lumbar BMD [36]. Overall, these data indicate that calcium supplements have a positive impact on BMD, particularly in individuals who adhere to the supplementation regimen and have a lower baseline dietary calcium intake.

An interesting finding of this study is that individuals of both sexes with a history of previous fragility fractures had significantly lower calcium intakes. The inverse correlation observed between dietary calcium intake and blood levels of PTH and β-CTX in the study population may suggest a physiological explanation for the potential protective effect of adequate calcium intake. However, the relationship between calcium intake and fracture risk remains complex and not yet well defined [37,38]. Undoubtedly, the literature agrees that an adequate dietary calcium intake is essential for achieving peak bone mass and supporting fracture healing [1,6]. Furthermore, calcium intake may have an adjunctive antisarcopenic effect alongside vitamin D, potentially reducing the risk of falls and, consequently, fractures [37]. Numerous meta-analyses of randomized controlled trials (RCTs) examining calcium, with or without vitamin D, for fracture prevention have been conducted. However, their conclusions often vary, despite analyzing largely the same studies. These discrepancies appear to arise primarily from differences in trial selection, definitions of fracture outcomes, and meta-analytic methodologies. Nevertheless, more recent meta-analyses consistently conclude that calcium supplementation with or without vitamin D, does not prevent hip or total fractures in community-dwelling individuals [39,40]. Recent longitudinal studies suggest that an increased consumption of dairy foods, which are rich in calcium and protein, may help reduce the risk of fragility fractures. In particular, a recent randomized controlled trial by Iuliano et al. demonstrated that, in institutionalized elderly individuals, increasing calcium intake through dairy foods, also rich in proteins, led to a significant reduction in falls and fractures, including hip fractures [38]. In two large U.S. cohorts—the Nurses’ Health Study (NHS) in women and the Health Professionals Follow-up Study (HPFS) in men—an additional daily serving of total dairy, with milk contributing about half, was associated with a statistically significant 6% reduction in hip fracture risk among postmenopausal women and men [41]. Furthermore, a more recent analysis of the NHS cohort found that consuming more than two servings of total dairy per day was associated with a lower risk of fractures, including hip fractures, compared to consuming less than one serving per day [42].

This study has some limitations. First, its cross-sectional design limits the ability to establish causal relationships. Second, the male sample size was smaller than that of the female participants. Nevertheless, this study also has notable strengths. First, dietary calcium intake was assessed using a food frequency questionnaire specifically validated for the Italian population. Second, as a single-center study, fracture assessment could be based directly on radiology reports, ensuring consistency. Finally, to our knowledge, this is the first study in Italy to examine the association between dietary calcium intake and body composition measured by DXA in an elderly population.

## 5. Conclusions

The results of this study suggest that an inadequate calcium intake remains highly prevalent in the Italian population, particularly among individuals with low BMD and a history of fractures. Additionally, in our study population, dietary calcium intake appears to influence body composition, showing an inverse association with body fat mass in women and a positive association with lean mass in men. An adequate dietary calcium intake, primarily from dairy foods rich in minerals and proteins, may help reduce the risk of falls and fractures.

## Figures and Tables

**Table 1:** General characteristics of the study population.

Variable	Women (*n* = 939)	Men (*n* = 173)
Age (yrs)	64.4 ± 6.4	65.1 ± 6.1
Weight (Kg)	65.6 ± 11.1	77.6 ± 11.0 ***
Height (cm)	160.5 ± 5.9	171.2 ± 6.5 ***
BMI (Kg/m^2^)	25.5 ± 4.2	26.4 ± 3.4 ***
Menopause Age (yrs)	49.5 ± 5.3	-
Creatinine (mg/dL)	0.89 ± 0.20	1.01 ± 0.26 *
Albumin (g/dL)	3.45 ± 0.37	3.51 ± 0.35
Calcium (mg/dL)	9.31 ± 0.51	9.12 ± 0.49 *
Phosphate (mg/dL)	3.47 ± 0.52	3.13 ± 0.58 *
ALP (UI/L)	82.61 ± 42.85	81.84 ± 34.65
25OHD (ng/mL)	23.47 ± 17.77	25.69 ± 13.62 *
PTH (pg/mL)	27.86 ± 14.86	24.60 ± 19.03 *
B-ALP (µg/L)	11.62 ± 6.25	9.87 ± 4.30 **
β-CTX (ng/mL)	0.613 ± 0.276	0.511 ± 0.289 **
LS-BMD (g/cm^2^)	0.986 ± 0.163	1.155 ± 0.187 ***
FN-BMD (g/cm^2^)	0.817 ± 0.116	0.904 ± 0.128 ***
TH-BMD (g/cm^2^)	0.881 ± 0.123	0.994 ± 0.142 ***
WB-BMD (g/cm^2^)	1.034 ± 0.106	1.153 ± 0.097 ***

* *p* < 0.05, ** *p* < 0.01, *** *p* < 0.001 for Student’s *t*-test of males vs. females.

**Table 2:** Age- and BMI-adjusted partial correlations of calcium intake with 25OHD, PTH, B-ALP, and β-CTX in male and female subjects.

	Calcium Intake (mg/day) in Males	Calcium Intake (mg/day) in Females
25OHD (ng/mL)	0.06	0.12 **
PTH (pg/mL)	−0.01	−0.09 *
B-ALP (µg/L)	−0.08	−0.05
β-CTX (ng/mL)	−0.24 *	−0.08 *

* *p* < 0.05, ** *p* < 0.01.

**Table 3:** Results of stepwise regression models to predict LS-BMD, FN-BMD, TH-BMD, and WB-BMD in males and females.

	Predictor Variable	b	95% CI	*p*
**Males**				
LS-BMD R^2^_adj_ = 0.16				
	BMI	0.021	0.011;0.031	0.001
FN-BMD R^2^_adj_ = 0.14				
	Calcium Intake	0.001	0.000;0.003	0.004
	BMI	0.010	0.003;0.017	0.017
TH-BMD R^2^_adj_ = 0.20				
	BMI	0.015	0.008;0.023	0.023
	Calcium Intake	0.002	0.001;0.003	0.001
WB-BMD R^2^_adj_ = 0.21				
	BMI	0.011	0.006;0.016	0.001
	Calcium Intake	0.001	−0.001;0.002	0.011
**Females**				
LS-BMD R^2^_adj_ = 0.16				
	BMI	0.010	0.008;0.013	0.001
	Age	−0.006	−0.008;−0.005	0.001
	History of Fracture	−0.024	−0.008;−0.005	0.049
FN-BMD R^2^_adj_ = 0.14				
	Age	−0.006	−0.007;−0.005	0.001
	BMI	0.008	0.006;0.010	0.001
TH-BMD R^2^_adj_ = 0.20				
	BMI	0.013	0.011;0.014	0.001
	Age	−0.005	−0.006;−0.004	0.001
	B-ALP	−0.002	−0.003;−0.001	0.004
WB-BMD R^2^_adj_ = 0.21				
	BMI	0.010	0.008;0.011	0.001
	Age	−0.005	−0.006;−0.004	0.001
	B-ALP	−0.002	−0.003;−0.001	0.001
	History of Fracture	−0.024	−0.040;−0.009	0.002
	Calcium Intake	0.010	0.007;0.018	0.026

The whole set of variables included in the model: BMI, age, B-ALP, 25OHD, PTH, β-CTX, calcium intake, and history of fracture. For all models, the significance value of the F statistic was <0.0001; Benjamini–Hochberg corrected *p* = 0.04. For each model, the predictors are listed in decreasing order according to the respective standardized values (β-coefficient) to indicate the decreasing relative contribution to the model.

## Data Availability

The data presented in this study are available on request from the corresponding author. The data are not publicly available due to privacy.

## Abbreviations

The following abbreviations are used in this manuscript:

BMD	Bone Mineral Density
FFQ	Food Frequency Questionnaire
PTH	Parathyroid Hormone
BMI	Body Mass Index
DXA	Dual-energy X-ray Absorptiometry
LS-BMD	Bone Mineral Density at Lumbar Spine
FN-BMD	Bone Mineral Density at Femoral Neck
TH-BMD	Bone Mineral Density at Total Hip
WB-BMD	Bone Mineral Density at Whole Body
FM	Fat Mass
LM	Lean Mass
25OHD	25-Hydroxyvitamin D
B-ALP	Bone Alkaline Phosphatase
βCTX	Type I Collagen β-Carboxy-Telopeptide
